# Cerebral amyloid deposition predicts long‐term cognitive decline in hemorrhagic small vessel disease

**DOI:** 10.1002/brb3.3189

**Published:** 2023-08-02

**Authors:** Ya‐Chin Tsai, Hsin‐Hsi Tsai, Chia‐Ju Liu, Sheng‐Sian Lin, Ya‐Fang Chen, Jiann‐Shing Jeng, Li‐Kai Tsai, Ruoh‐Fang Yen

**Affiliations:** ^1^ Department of Nuclear Medicine National Taiwan University Hospital Hsin‐Chu Branch Hsinchu Taiwan; ^2^ Department of Neurology National Taiwan University Hospital Bei‐Hu Branch Taipei Taiwan; ^3^ Department of Neurology National Taiwan University Hospital Taipei Taiwan; ^4^ Department of Nuclear Medicine National Taiwan University Hospital Taipei Taiwan; ^5^ Department of Medical Imaging National Taiwan University Hospital Taipei Taiwan; ^6^ Department of Neurology National Taiwan University Hospital Hsin‐Chu Branch Hsinchu Taiwan

**Keywords:** cognitive impairment, dementia, intracerebral hemorrhage, Pittsburgh compound B, small vessel disease

## Abstract

**Background:**

To investigate the association between cerebral amyloid deposition and long‐term cognitive outcomes in patients with hemorrhagic small vessel disease (SVD) and survivors of intracerebral hemorrhage (ICH).

**Methods:**

Patients experiencing an ICH without overt dementia were prospectively recruited (*n* = 68) for brain MRI and Pittsburgh compound B (PiB) positron emission tomography scans at baseline. Cognitive function was assessed using the mini‐mental status examination (MMSE) and clinical dementia rating after an overall median follow‐up of 3.8 years. A positive amyloid scan was defined as a global PiB standardized uptake value ratio >1.2. Associations between follow‐up cognitive outcomes and neuroimaging markers were explored using multivariable Cox regression models.

**Results:**

PiB(+) patients were older (72.1 ± 7.8 vs. 59.9 ± 11.7, *p* = .002) and more frequently had cerebral amyloid angiopathy (CAA) (63.6% vs. 15.8%, *p* = .002) than PiB(−) patients. PiB(+) was associated with a higher risk of dementia conversion (32.9 vs. 4.0 per 100‐person‐years, hazard ratio [HR] = 15.7 [3.0–80.7], *p* = .001) and MMSE score decline (58.8 vs. 9.9 per 100‐person‐years, HR = 6.2 [1.9–20.0], *p* = .002). In the non‐CAA subgroup (*n* = 52), PiB(+) remained an independent predictor of dementia conversion, *p* = .04). In the Cox models, PiB(+) was an independent predictor of dementia conversion (HR = 15.8 [2.6–95.4], *p* = .003) and MMSE score decline (HR = 5.7 [1.6–20.3], *p* = .008) after adjusting for confounders.

**Conclusions:**

Cerebral amyloid deposition potentially contributes to long‐term cognitive decline in SVD‐related ICH.

## Graphical abstract

This study with 68 dementia‐free small vessel disease‐related intracerebral hemorrhage survivors investigated the association between cerebral amyloid deposition and long‐term cognitive outcomes.We found cerebral amyloid depositions are strong indicators of long‐term cognitive decline in both patients with cerebral amyloid angiopathy and hypertensive arteriosclerosis.

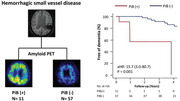

## 1 INTRODUCTION

Intracranial hemorrhage (ICH) accounts for approximately one quarter of strokes and has severe consequences of high morbidity and mortality (Qureshi et al., 2001). The majority of patients who suffer ICH exhibit cognitive impairment in the acute phase; however, the long‐term cognitive trajectories are highly variable (Aam et al., 2020; Banerjee et al., 2018). Cognitive impairment or even dementia commonly occurs in survivors of ICH (Planton et al., 2017); however, studies of poststroke cognitive impairment have predominantly been performed in survivors of ischemic stroke, with survivors of ICH only comprising small subsets of the study cohorts (Rost et al., 2022). The largest single‐center prospective cohort study of dementia‐free ICH survivors conducted to date reported the incidence of dementia was 14% at 1‐year post ICH, and this rate had doubled by 4 years (Moulin et al., 2016). The development of progressive cognitive dysfunction after spontaneous ICH may represent a distinct entity as these patients may intrinsically harbor cerebral small vessel disease (SVD), which predisposes them to long‐term neurocognitive disorders (Shi & Wardlaw, 2016).

Preexisting parenchymal damage due to underlying microangiopathy is considered to be a major mechanism underlying the development of post‐ICH cognitive impairment (Benedictus et al., 2015; Pasi et al., 2021). Among the different types of SVD, cerebral amyloid angiopathy (CAA) and related markers have been closely associated with the risk of cognitive decline (Moulin et al., 2016; Potter et al., 2021), which suggests that amyloid may be a key player in the cognitive changes observed in survivors of ICH. However, due to a lack of biomarkers to directly measure cerebral amyloid load, the relationship between amyloid and the development of cognitive impairment in this specific population remains unclear. To our knowledge, no study has yet assessed the association between cerebral amyloid deposition and the long‐term cognitive outcomes of survivors of spontaneous ICH with hemorrhagic SVD pathology.

Amyloid positron emission tomography (PET) is a noninvasive method for identifying CAA and β‐amyloid deposition in survivors of spontaneous ICH (Baron et al., 2014; Gurol et al., 2016; Ly et al., 2010). Previous studies showed that the retention signals of amyloid PET in survivors of ICH appear to correlate with CAA‐related neuroimaging markers, including lobar hemorrhagic lesions, white matter hyperintensities (WMH), and the severity of centrum semiovale perivascular spaces (CSO‐PVS) (Gurol et al., 2013; Raposo et al., 2019; Tsai et al., 2017, 2021). This evidence implies that tracer retention could be employed as a surrogate marker for vascular amyloid burden. However, current amyloid ligands also bind to parenchymal Aβ plaques; therefore, the retention signal could also suggest underlying Alzheimer's pathology, especially when there is a substantial overlap between AD and CAA (Greenberg et al., 2020).

In this prospective cohort study, we included survivors of ICH without overt dementia who underwent a cerebral amyloid scan to compare the longitudinal cognitive outcomes of patients with positive and negative amyloid scans. As a secondary objective, we investigated the independent associations between amyloid deposition and cognitive outcomes using a Cox regression model that included other neuroimaging markers as covariates. Our hypothesis and the major aim of this study were to confirm that a positive amyloid scan is an independent predictor of cognitive decline in survivors of ICH with SVD.

## 2 METHODS

### 2.1 Data availability

All data from this article are being held within National Taiwan University Hospital (NTUH) and will be shared with qualified investigators on request.

### 2.2 Patient enrollment

**FIGURE 1:**
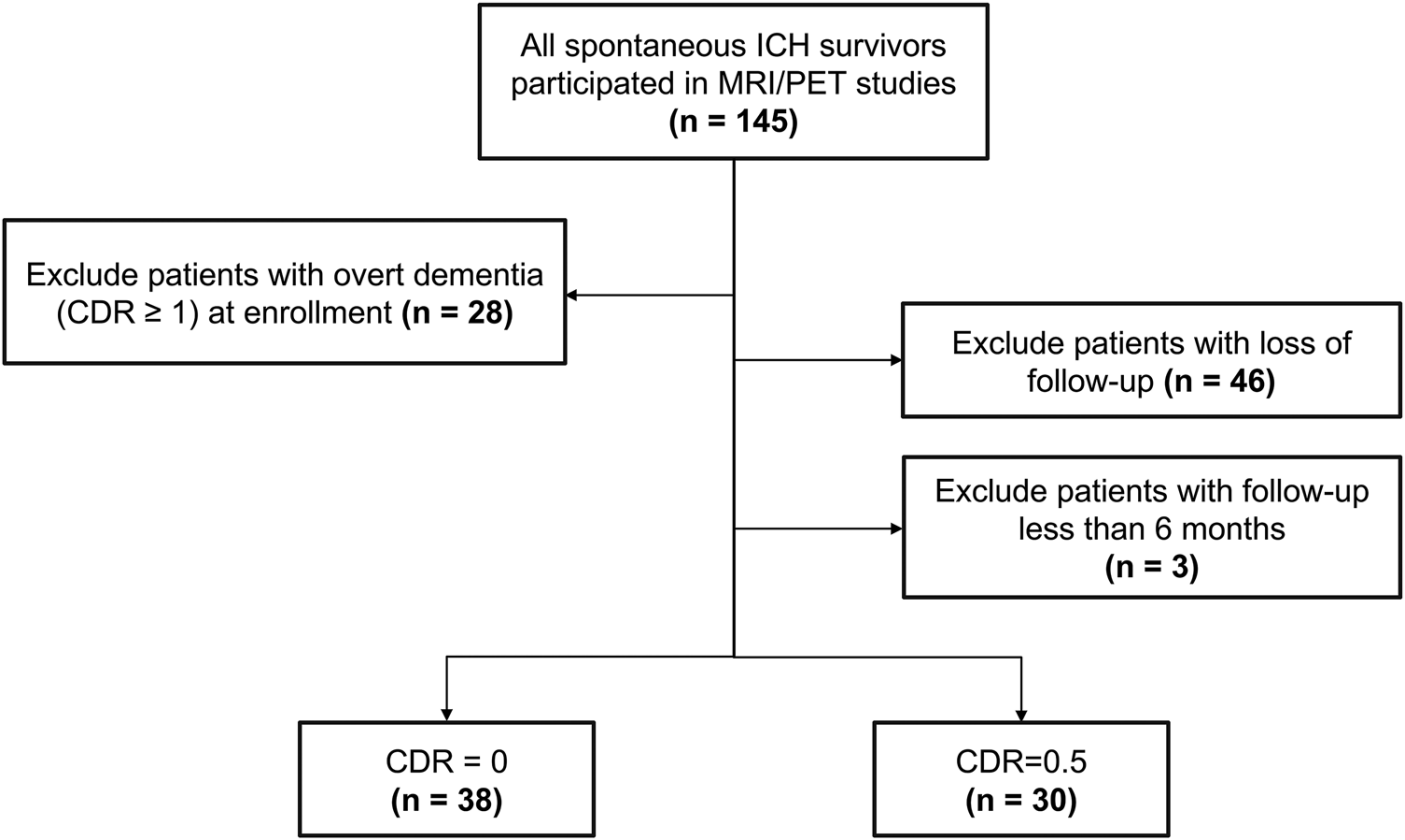
Flowchart of patient enrollment.

We prospectively recruited patients aged ≥50 years old who suffered a symptomatic spontaneous ICH and underwent brain MRI and ^11^C‐Pittsburgh compound B (PiB) PET scans at NTUH from September 2014 to October 2021 (Tsai et al., 2020, 2021). Patients with potential secondary causes, including trauma, structural lesions, brain tumors, or coagulopathy, and patients who had an infarct with hemorrhagic transformation were excluded. A total of 145 survivors of ICH who were diagnosed with intrinsic SVD and fulfilled the enrollment criteria agreed to participate in this study and underwent brain MRI and PiB PET scans (Figure 1). We excluded patients who had overt dementia (clinical dementia rating [CDR] ≥1) at enrollment (*n* = 28). Patients who did not complete the follow‐up cognitive assessment (*n* = 46) and patients with less than 6 months of follow‐up (*n* = 3) were also excluded. A final sample of 68 patients were included in this analysis. Baseline clinical data were collected by the investigators through a comprehensive review of medical records and interviewing each participant. We collected data on the following demographic characteristics: age, sex, years of education, diagnoses of chronic hypertension, diabetes or hypercholesterolemia, and renal function (represented by the estimated glomerular filtration rate). At enrollment, cognitive status was assessed in each patient through a combination of history taking and an objective cognitive assessment that included the mini‐mental state examination (MMSE) and CDR scores on the day the patients underwent the PiB PET scan. The median interval between ICH onset and PET scan/baseline cognitive assessment was 12 (interquartile range 3–40) months.

### 2.3 Cognition follow‐up data

The cognitive status of each patient was followed‐up at our neurology clinic during their regular visits; the cognitive evaluations were performed as part of routine clinical protocols by neuropsychologists each year. The diagnosis of dementia was based on the National Institute on Aging‐Alzheimer's Association criteria for all‐cause dementia (McKhann et al., 2011), which requires the patient to present cognitive or behavioral symptoms that interfere with their ability to perform functions at work or their usual activities, that represent a decline from previous levels, and that cannot be explained by delirium or a major psychiatric disorder. For the purpose of this study, patients without cognitive issues who did not undergo a cognitive assessment at the clinic were asked to complete a formal interview with our neuropsychologist between November 2021 and May 2022 to confirm their cognitive status (*n* = 21, 30.9% of analyzed patients). Patients were followed‐up from their date of enrollment (i.e., the date of the PiB scan) until the occurrence of dementia or the date of last cognitive assessment. Additionally, recurrent strokes, including ICH and incident ischemic stroke events, were also recorded for each patient in order to determine whether these events were related to cognitive decline.

### 2.4 MRI acquisition and analysis

Brain MRIs were obtained using a 3‐Tesla scanner (Siemens Verio, TIM or mMR, Siemens Medical Solutions). The imaging protocols included T1‐weighted imaging, T2‐weighted imaging, fluid‐attenuated inversion recovery imaging, susceptibility weighted imaging (SWI), diffusion‐weighted imaging, and apparent diffusion coefficient maps, as previously described (Tsai et al.,^2020^ , ^2021^). According to the Boston criteria V2.0 (Charidimou et al., 2022), patients with lobar ICH(s) involving the cerebral cortex and underlying white matter with strictly lobar cerebral microbleeds (CMBs), cortical superficial siderosis (cSS), or white matter features (severe CSO‐PVS or WMH in a multisport pattern) were defined as having probable (*n* = 11) or possible (*n* = 5) CAA; all other cases that had deep ICH or deep CMB(s) were defined as non‐CAA ICH (*n* = 52). These patients were presumed to have predominant hypertensive deep perforator arteriopathy (Pasi et al., 2018; Tsai et al., 2019b).

MRI markers related to cerebral SVD were evaluated based on the Standards for Reporting Vascular Changes on neuroimaging criteria (STRIVE) (Tsai et al., 2019b, 2021; Wardlaw et al., 2013). The readers were blinded to patient identity, clinical diagnosis, and other study results prior to the evaluation. Briefly, the number of CMBs and the presence of cSS were evaluated using axial SWI sequences (Charidimou et al., 2017; Greenberg et al., 2009). The number of CMBs in the lobar (i.e., the frontal, temporal, parietal, occipital, and insular cortices) and deep regions (i.e., the brainstem, basal ganglia [BG], thalamus, internal capsule, external capsule, corpus callosum, and deep periventricular white matter) were counted. Lacunes were evaluated in the supratentorial region and defined as “round or ovoid, subcortical, fluid‐filled cavities, from 3 to 15 mm in diameter,” and supratentorial lacunes were classified as lobar or deep lacunes based on the definition in previous studies (Pasi et al., 2017; Tsai et al., 2018). The severity of WMH was rated using the Fazekas scale in both the periventricular and deep brain regions (Kim et al., 2008); the highest score was considered the overall WMH score for each patient. WMH volume was calculated based on fluid‐attenuated inversion recovery imaging of the ICH‐free hemisphere and multiplied by two, as we previously proposed (Tsai et al., 2021). MRI‐visible PVS were evaluated on T2‐weighted imaging and defined as sharply delineated structures measuring <3 mm following the course of perforating or medullary vessels (Charidimou et al., 2017). High‐degree PVS was defined as greater than >20 visible PVS in the CSO or in the BG on the side of the brain with more severe involvement (Charidimou et al., 2017; Doubal et al., 2010).

### 2.5 PET acquisition and analysis

PiB was manufactured and handled according to good manufacturing practice at the PET Center, NTUH (specific activity: 39 ± 19 GBq/μmol). PET scanning was performed within 3 months after MRI. Static PET/CT scans (Discovery ST; GE Healthcare) were acquired in 3‐dimensional mode for 30 min beginning 40 min postinjection of 10 mCi ^11^C‐PiB. PET data were reconstructed via ordered set expectation maximization (5 iterations, 32 subsets, post filter 2.57) and corrected for attenuation. Each PiB PET image was realigned, resliced, and manually co‐registered to a standardized CT template using PMOD software (Tsai et al., 2019a, 2019a, 2021).

**FIGURE 2:**
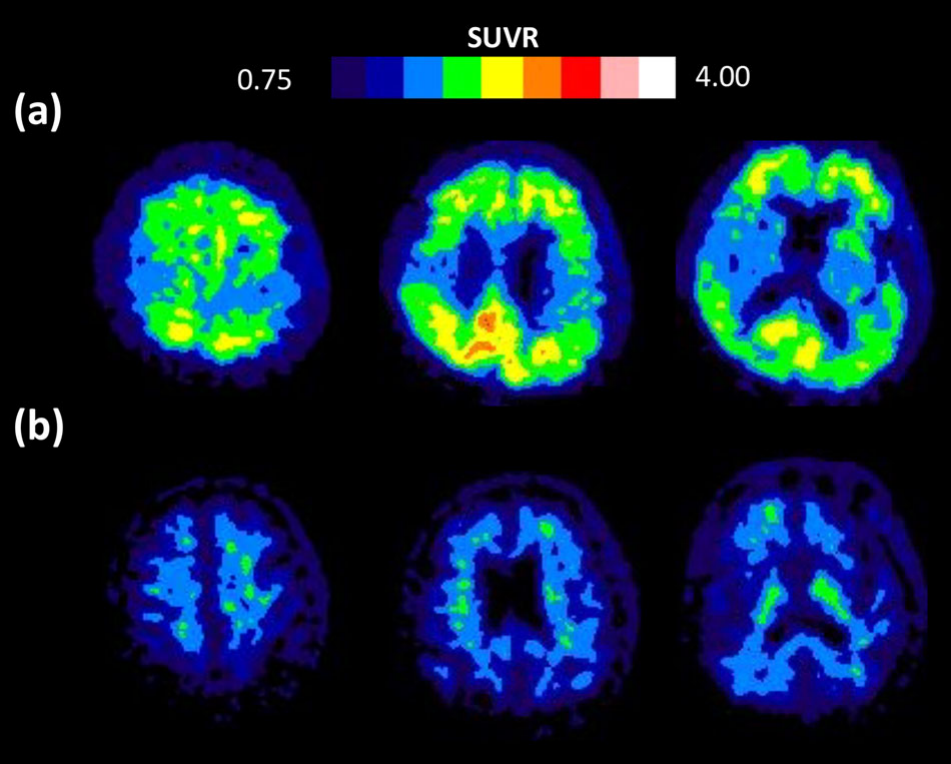
**Representative positive and negative Pittsburgh compound B (PiB) scans**. We defined amyloid scan positivity based on the global PiB uptake standardized uptake value ratio (SUVR) using the cutoff value of 1.2: (a) example of a positive PiB scan with a global SUVR of 1.9 and (b) a negative PiB scan with a global SUVR of 0.9.

PET data werexpressed as the average mean standardized uptake value ratio (SUVR) of the whole cortex using the cerebellar cortex as a reference region. Areas of macrobleeds were manually excluded from SUVR analyses. A positive amyloid PET was operationally defined as a SUVR >1.2 based on the criterion in our previous report (Tsai et al., 2017). Representative PiB(+) and PiB(−) scans are shown in Figure 2.

### 2.6 Statistical analysis

We compared the baseline demographic information and neuroimaging variables of the patients with PiB(+) and PiB(−) scans. Discrete variables are presented as counts (%), and continuous variables are presented as mean (±standard deviation) or median (interquartile range), as appropriate, based on their distribution. Categorical variables were analyzed using Fisher's exact test, and continuous variables were analyzed using the Mann–Whitney *U*‐test. The follow‐up time for each patient was calculated from the date of the PiB scan until the date of dementia conversion or last clinical documentation of a cognitive assessment. The rates of dementia conversion and MMSE score decline (defined as a MMSE score decline ≥2 points) were determined from the incidence per person‐years. Kaplan–Meier analyses were conducted to plot the disease‐free probability. Cox regression analyses were performed to calculate the hazard ratios (HRs) and 95% confidence intervals for the occurrence of dementia and MMSE score decline for the patients stratified by clinical and radiological markers, including amyloid scan positivity, primary ICH location (lobar vs. non‐lobar), ICH etiology (CAA vs. non‐CAA), number of lobar CMBs (>5 vs. ≤5), cSS (presence vs. absence), WMH (moderate‐to‐severe vs. none‐to‐mild), lacunes (presence vs. absence), CSO‐PVS (high‐ vs. low‐degree), and BG‐PVS (high‐ vs. low‐degree). The analyses were also adjusted for age, sex, years of education, and baseline CDR (0 or 0.5). Further Cox regression models were also built to evaluate the differences in the follow‐up outcomes of PiB(+) and PiB(−) patients. In addition to age, sex, years of education, and baseline CDR, the following variables were entered as covariates: number of lobar CMBs (>5 vs. ≤5), lacunes (presence vs. absence), and CSO‐PVS (high‐ vs. low‐degree) due to their potential associations with cognitive outcomes in the previous analysis (*p* < .2). Variance inflation factors were used to test the collinearity between the variables; the variance inflation factors for all of the tested neuroimaging markers (PiB positivity, number of lobar CMBs, lacunes, CSO‐PVS) were less than 1.2. For each Cox regression model, a time‐dependent covariate was generated by creating an interaction of the predictor and a function of survival time. The time‐dependent covariate was then included in the original model to verify the proportional hazard assumption. All of the time‐dependent covariates in our testing models were nonsignificant. Statistical analyses were performed using SPSS version 25 (SPSS Inc.). All tests of significance were two‐tailed and based on a threshold for significance of *p* < .05.

### 2.7 Standard protocol approvals, registrations, and patient consent

This study was performed with the approval of the institutional review board (201903069RINB) of NTUH and in accordance with their guidelines. Written informed consent was obtained from all participants or their family members.

## 3 RESULTS

Of the 145 patients who agreed to participate in this study, 77 participants were excluded from further analysis (Figure 1). The excluded patients were older (69.4 ± 11.1 vs. 61.9 ± 12.0 years, *p* < .001), had fewer years of education (9.6 ± 4.0 vs. 12.4 ± 4.0, *p* < .001), and exhibited lower performance in the MMSE (20.9 ± 8.5 vs. 27.2 ± 3.6, *p* < .001). The excluded patients were also more frequently diagnosed with probable CAA (35.1% vs. 16.2%, *p* = .013), had a higher prevalence of positive amyloid scans (46.8% vs. 16.2%, *p* < .001), and exhibited more severe SVD markers, as shown in Table S1. Of the 68 patients included in this analysis, 35 patients (51.5%) were survivors of lobar ICH, and 33 patients (48.5%) were survivors of non‐lobar ICH. The overall median follow‐up time was 45 months (interquartile range, 24–75 months). Sixteen patients (23.5% of the entire cohort; 45.7% of the lobar ICH subgroup) were diagnosed with CAA (eleve probable CAA; five possible CAA) based on the Boston criteria V2.0. Eleven patients (16.2%, seven in the CAA subgroup and four in the non‐CAA subgroup) exhibited positive amyloid deposition on PiB PET (defined as a global SUVR >1.2).

The comparisons of the patients with positive and negative amyloid scans are shown in Table 1. Patients with PiB(+) scans were significantly older (mean age, 72.1 ± 7.8 vs. 59.9 ± 11.7, *p* = .002), had fewer years of education (10.3 ± 3.7 vs. 12.8 ± 4.0, *p* = .045), and more frequently had mild cognitive impairment at enrollment (CDR 0.5, 81.8% vs. 36.8%, *p* = .008) than patients with PiB(−) scans. Patients with PiB(+) scans more frequently had CAA (63.6% vs. 15.8%, *p* = .002) than patients with PiB(−) scans; however, other neuroimaging markers were similar between the two groups, including lobar and deep CMBs, WMH volume, lacunes, MRI‐visible PVS, cSS, and hippocampal volume (all *p* > .05).

**TABLE 1:** Comparison of the demographics of patients with positive and negative amyloid scans.

	PiB PET (+) (*n* = 11)	PiB PET (−) (*n* = 57)	*p‐*Value
Male, %	7 (63.6%)	38 (66.7%)	>.999
**Age, years**	**72.1 ± 7.8**	**59.9 ± 11.7**	**.002**
**Years of education**	**10.3 ± 3.7**	**12.8 ± 4.0**	**.045**
Hypertension, %	8 (72.7%)	51 (89.5%)	.154
Diabetes, %	1 (9.1%)	10 (17.5%)	.677
Hypercholesterolemia, %	2 (18.2%)	19 (33.3%)	.482
EGFR, mL/min	81.9 ± 28.8	90.2 ± 22.5	.156
**Baseline CDR 0.5, %**	**9 (81.8%)**	**21 (36.8%)**	**.008**
MMSE	26.8 ± 3.7	27.3 ± 3.6	.318
Lobar/non‐lobar ICH	8/3 (72.7/27.3%)	27/30 (47.4/52.6%)	.189
Frontal/temporal/parietal/occipital lobe	3/0/5/0	4/7/12/4	
BG/thalamus/infratentorial region	2/1/0	20/9/1	
Interval between ICH and PET, months	33 (4–81)	10 (3–39)	.338
**CAA**	**7 (63.6%)**	**9 (15.8%)**	**.002**
**Probable CAA**	**6 (54.5%)**	**5 (8.8%)**	**.001**
Cerebral microbleed			
Lobar CMBs (+)	7 (63.6%)	37 (64.9%)	>.999
Number of lobar CMBs	3.6 ± 4.6	6.3 ± 14.5	.939
Deep CMBs (+)	3 (27.3%)	32 (56.1%)	.105
Number of deep CMBs	3.5 ± 7.6	6.3 ± 14.5	.184
White matter hyperintensities			
Fazekas scale ≥2	9 (81.8%)	31 (54.4%)	.108
Volume, mL	18.8 ± 9.6	13.8 ± 14.5	.108
Lacunes, %	4 (36.4%)	26 (45.6%)	.743
Lobar lacune, %	4 (36.4%)	21 (36.8%)	>.999
Deep lacune, %	1 (9.1%)	13 (22.8%)	.437
MRI‐visible enlarged perivascular spaces			
Basal ganglia (>20), %	6 (54.5%)	28 (49.1%)	1.000
Centrum semiovale (>20), %	5 (45.5%)	17 (29.8%)	.316
Cortical superficial siderosis, %	2 (18.2%)	2 (3.5%)	.120
Hippocampal volume, mm^3^	3660 ± 482	3780 ± 706	.590
**Global PiB SUVR (IQR)**	**1.53 (1.23–1.60)**	**1.04 (1.01–1.09)**	**<.001**

*Note*: Values are mean (±standard deviation), median (IQR), or number (percentage). Significant differences are shown in bold.

Abbreviations: CAA, cerebral amyloid angiopathy; CMB, cerebral microbleeds; IQR, interquartile range; MMSE, mini‐mental status exam; PiB, Pittsburgh compound B; SUVR, standardized uptake value ratio;.

**FIGURE 3:**
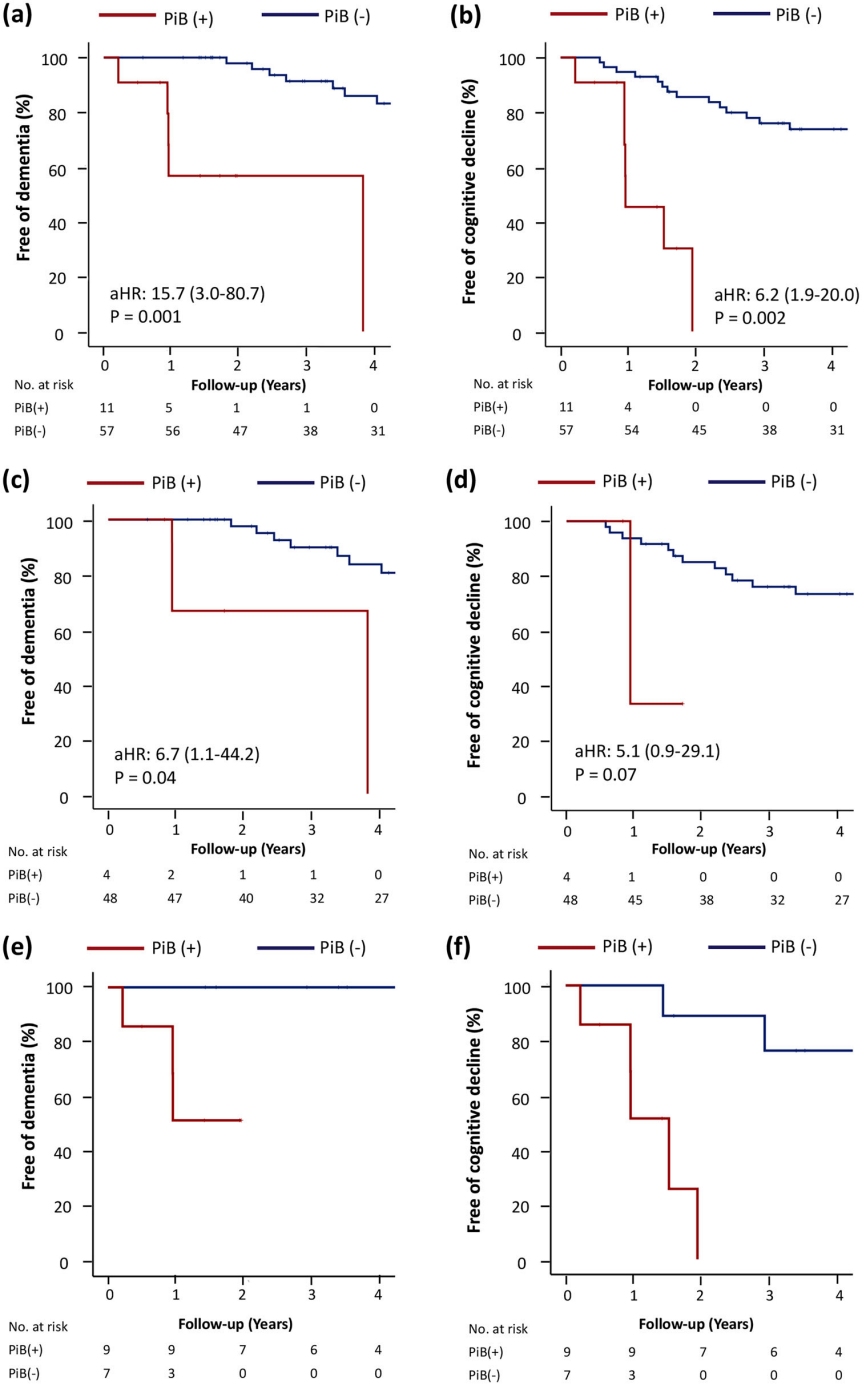
**Kaplan–Maier curves for cognitive change in survivors of intracerebral hemorrhage**: (a) dementia conversion for the entire cohort with Pittsburgh compound B (PiB)(+) and PiB(−) scans, (b) cognitive decline (mini‐mental status examination [MMSE] decline ≥2) for the entire cohort with PiB(+) and PiB(−) scans, (c) dementia conversion for non‐cerebral amyloid angiopathy (CAA) patients with PiB(+) and PiB(−) scans, (d) cognitive decline for non‐CAA patients with PiB(+) and PiB(−) scans, (e) dementia conversion for CAA patients with PiB(+) and PiB(−) scans, and (f) cognitive decline for CAA patients with PiB(+) and PiB(−) scans.

The event rates for dementia conversion and MMSE score decline in the entire cohort stratified by the neuroimaging markers are shown in Table 2. The Kaplan–Maier curves for cognitive change in the patients with PiB PET(+) and PiB(−) scans are shown in Figure 3 (entire cohort: a and b; non‐CAA ICH: c and d; CAA‐ICH: e and f). PiB(+) was associated with a higher risk of dementia conversion (32.9 vs. 4.0 per 100‐person‐years, HR = 15.7 [3.0–80.7], *p* = .001) and MMSE score decline (58.8 vs. 9.9 per 100‐person‐years, HR = 6.2 [1.9–20.0], *p* = .002) compared to PiB(−) after adjustment for age, sex, years of education, and baseline CDR (Figure 3a,b). When only non‐CAA patients were analyzed (*n* = 52), PiB(+) remained associated with dementia conversion (HR = 6.7 [1.1–44.2], *p* = .04, Figure 3c) and a strong trend toward MMSE score decline (HR = 5.1 [.9–29.1], *p* = .07; Figure 3d). Additionally, five patients developed recurrent ICH (three lobar and two deep ICHs) and two patients developed incident ischemic stroke during follow‐up. PiB(+) was not significantly associated with a higher risk of recurrent stroke than PiB(−) after adjustment for age and sex (HR = 1.3 [.2–7.1], *p* = .765). Only one patient exhibited a stepwise cognitive change related to a recurrent ICH episode.

**TABLE 2:** Event rates and Cox regression model of the associations between neuroimaging markers and cognitive change.

	Dementia conversion			MMSE decline ≥2		
	Rate (% per person‐years)	HR (95% CI)	*p*‐Value	Rate (% per person‐years)	HR (95% CI)	*p*‐Value
**Amyloid scan**						
**PiB scan (+) (*n* = 11)**	**32.9**	**15.7 (3.0–80.7)**	**.001**	**58.8**	**6.2 (1.9–20.0)**	**.002**
**PiB scan (**−**) (*n* = 57)**	**4.0**	**1.0**		**9.9**		
Primary ICH location						
Lobar (*n* = 35)	4.7	.8 (.2–3.5)	.717	12.7	.9 (.4–2.1)	.866
Non‐lobar (*n* = 33)	6.6	1.0		11.6	1.0	
ICH etiology						
CAA ICH (*n* = 16)	6.9	1.6 (.4–7.4)	.521	16.3	1.0 (.4–2.4)	.947
Non‐CAA ICH (*n* = 52)	5.5	1.0		11.3	1.0	
Number of lobar CMBs						
>5 (*n* = 18)	12.0	1.9 (.6–6.3)	.292	19.4	2.1 (.9–5.0)	.103
≤5 (*n* = 50)	3.6	1.0		9.9	1.0	
Cortical superficial siderosis						
Presence (*n* = 4)	11.6	3.2 (.3–34.3)	.332	24.4	2.5 (.5–10.3)	.297
Absence (*n* = 64)	5.5	1.0		11.8	1.0	
Overall WMH						
Moderate‐to‐severe (*n* = 40)	9.3	3.2 (.5–19.7)	.215	16.3	.9 (.3–2.1)	.684
None‐to‐mild (*n* = 28)	1.6	1.0		7.5	1.0	
Lacunes						
Presence (*n* = 30)	5.4	1.2 (.2–6.4)	.818	14.0	2.5 (.9–6.8)	.079
Absence (*n* = 38)	6.0	1.0		10.8	1.0	
**CSO‐PVS**						
**High degree (*n* = 22)**	1.4	1.1 (.1–10.2)	.934	**14.1**	**2.4 (1.0–5.5)**	**.048**
**Low degree (*n* = 46)**	7.3	1.0		**11.4**	**1.0**	
BG‐PVS						
High degree (*n* = 34)	9.2	1.4 (.4**–**4.8)	.658	17.8	1.4 (.6**–**3.0)	.507
Low degree (*n* = 34)	2.8	1.0		7.7	1.0	

*Note*: All Cox models were adjusted for age, sex, years of education, and baseline CDR. Significant factors are shown in bold.

Abbreviations: BG, basal ganglia; CAA, cerebral amyloid angiopathy; CDR, clinical dementia rating; CSO, centrum semiovale; ICH, intracerebral hemorrhage; CMB, cerebral microbleed; MMSE, mini‐mental status exam; PVS, perivascular spaces; WMH, white matter hyperintensity.

In the entire cohort, a high degree CSO‐PVS was also associated with MMSE score decline (HR = 2.4 [1.0–5.5], *p* = .048) but not dementia conversion (*p* = .934; Table 2). Patients with a higher number of lobar CMBs (*p* = .103) and the presence of lacune (*p* = .079) exhibited a trend toward MMSE score decline. The other neuroimaging markers, including primary ICH location (lobar vs. non‐lobar), CAA diagnosis, cSS, WMH severity, and BG‐PVS, were not associated with dementia conversion or MMSE score decline (all *p* > .05).

Next, we built the multivariable Cox regression models to investigate whether amyloid positivity independently predicts cognitive outcomes (Table 3). Lobar CMB, lacune, and CSO‐PVS were included in the model as they were potentially associated with cognitive worsening in previous analyses. In this model, age showed a strong trend toward a prediction of dementia conversion (HR = 2.2 [.9–5.0], *p* = .066) and was significantly associated with MMSE score decline (HR = 2.2 [1.3–3.8], *p* = .003). PiB(+) remained an independent predictor of dementia conversion (HR = 15.8 [2.6–95.4], *p* = .003) and MMSE score decline (HR = 5.7 [1.6–20.3], *p* = .008; Table 3).

**TABLE 3:** Multivariable Cox regression models of factors that predict cognitive outcomes among survivors of intracerebral hemorrhage.

	Dementia conversion			MMSE decline ≥2		
	HR	95% CI	*p‐*Value	HR	95% CI	*p‐*Value
Age, per 10 years	2.2	.9**–**5.0	.066	2.2	1.3**–**3.8	.003
PiB PET (+)	15.8	2.6**–**95.4	.003	5.7	1.6**–**.3	.008
>5 Lobar CMBs	1.5	.3**–**6.2	.604	2.2	.8**–**5.5	.113
Presence of lacunes	.6	.1**–**5.4	.653	2.3	.8**–**7.0	.136
High‐degree CSO‐PVS	.8	.1**–**12.4	.899	2.1	.8**–**5.7	.133

*Note*: The Cox model was also adjusted for sex, years of education, and baseline CDR.

Abbreviations: BG, basal ganglia; CAA, cerebral amyloid angiopathy; CDR, clinical dementia rating; CMB, cerebral microbleeds; CSO, centrum semiovale; ICH, intracerebral hemorrhage; CMB, cerebral microbleed; MMSE, mini‐mental status exam; PiB, Pittsburgh compound B; PVS, perivascular spaces; WMH, white matter hyperintensity.

## 4 DISCUSSION

We report the cognitive follow‐up outcomes for a cohort of survivors of SVD‐related ICH who were not demented at enrollment. We prospectively investigated the associations between cerebral amyloid deposition and the long‐term risk of dementia conversion and MMSE score decline using cerebral PiB scans. We show that amyloid scan positivity is associated with a risk of cognitive decline in survivors of ICH. A positive amyloid scan was independently associated with a 33% incidence per person‐years and 16‐fold higher risk of dementia conversion. These associations were far more significant than the effects of other radiological markers of SVD parenchymal injuries on MRI. Even among survivors of ICH with presumed microangiopathy due to hypertensive arteriopathy, a positive amyloid scan remained a strong predictive factor for cognitive decline, including dementia conversion and MMSE score decline. Overall, our findings imply that cerebral amyloid pathology, which can be related to vascular or parenchymal β‐amyloid, is a potential driving factor for cognitive decline in patients harboring hemorrhagic SVD.

The current evidence suggests that preexisting underlying microangiopathy and the related accumulation of parenchymal injuries contribute substantially to the long‐term cognitive trajectory of survivors of spontaneous ICH (Biffi et al., 2016; Pasi et al., 2021; Xiong et al., 2019). As hypertensive arteriopathy and CAA are the most common sporadic types of microangiopathy in ICH, several factors associated with CAA have been identified as predictors of post‐ICH cognitive impairment, including age, the *ApoE* ε4 allele, lobar hematoma, and cSS (Moulin et al., 2016; Potter et al., 2021). Our findings also support this observation, as a positive amyloid scan—which has been proposed as a neuroimaging marker of CAA (Chen et al., 2019)—independently predicted the development of post‐ICH dementia conversion and MMSE score decline. One of the most important findings in this study is the discovery that amyloid deposition is a stronger predictor of cognitive decline than the ischemic or hemorrhagic insults due to CAA, such as lobar CMB or WMH. This observation implicates that amyloid plaques on either the vessel walls (as in CAA) or in the parenchyma (as in Alzheimer's disease) potentially play a role in driving the neurodegenerative process in hemorrhagic SVD. The presence of severe CSO‐PVS was the only MRI marker that was significantly associated with cognitive decline in this study. This finding is in line with several other studies performed in longitudinal aging cohorts (Ding et al., 2017; Paradise et al., 2021; Zhu et al., 2010), and we extends this observation into survivors of ICH. Although the fact that some evidence suggests severe CSO‐PVS has been related to cerebral amyloid pathology and may represent a potential marker of impairment to the brain waste clearance system (Charidimou et al., 2022; Mestre et al., 2017; Tsai et al., 2021), the exact pathophysiology for the development of CSO‐PVS and its clinical impact still remain controversial. The relationships among CSO‐PVS, amyloid deposition, and cognitive decline in survivors of ICH merit investigation in future studies.

A previous study by our group demonstrated that concomitant CAA and higher amyloid load observed on PET could be found in a subgroup of patients with mixed‐ICH, namely, patients with hemorrhagic lesions involving both the lobar and deep regions (Tsai et al., 2019a). In our current cohort, 8% of patients with non‐CAA ICH (i.e., strictly deep ICH/CMBs or mixed lobar and deep ICH/CMBs) had positive PiB scans. Due to the limitation of concurrent tracer binding to distinguish between vascular and parenchymal amyloid deposition, a positive amyloid PET scan suggests either concomitant CAA or overlapping AD pathology in these patients who had presumed hypertensive arteriopathy. One important impact of the current study is the finding that a positive amyloid scan remains an important determinant for cognitive outcomes in non‐CAA ICH. This result implies that the identification of cerebral amyloid deposition is critical, even in patients who have CAA that does not meet the Boston criteria—especially in Asian cohorts where the cerebral pathology of deep perforator arteriopathy appears to be more frequent than in Caucasian populations (Yakushiji et al., 2019).

The observation of elevated dementia risk in CAA has been attributed to a neuronopathic process related to vascular pathology (Boyle et al., 2015; Smith, 2018; Vasilevko et al., 2010). As CAA is an Aβ‐related disease, it is unclear whether the risk of cognitive decline is related to amyloid deposition itself or the accumulation of vascular dysfunction (Gokcal et al., 2022). Interestingly, we did not find a higher dementia risk in patients with CAA defined using the Boston criteria V2.0, which is based on MRI‐related hemorrhagic insults and white matter markers. This observation also suggests that parenchymal amyloid pathology may impose a significant effect on the cognitive decline in hemorrhagic SVD. This result raises an interesting research question related to the contribution of tau protein, as tau is a frequent concomitant pathology in Aβ‐related disorders. Unfortunately, we were unable to assess the effects of tau in the current cohort. Longitudinal investigations of the cerebral tau load in patients with coexisting SVD and positive amyloid scans could provide further insight into the complex relationship between vascular disease and Alzheimer's pathology and their interactive effect on cognitive outcomes.

Positive cerebral amyloid scans can predict the conversion from mild cognitive impairment to Alzheimer's dementia in memory cohorts (Frings et al., 2018; Trzepacz et al., 2014) and have been associated with more rapid cognitive decline in patients after stroke and transient ischemic attack (Liu et al., 2015). To our knowledge, this is the first study to specifically demonstrate a relationship between amyloid PET scan status and cognitive outcomes in survivors of ICH. Cognitive impairment commonly occurs after SVD‐related ICH (Planton et al., 2017) and is a significant factor for long‐term disability in these patients. However, there is a lack of useful markers to predict the risk of dementia in survivors of ICH. Our results clinically imply that cerebral amyloid scans may have potential as an indicator of cognitive outcomes in survivors of SVD‐related ICH. The cognitive function of patients with evidence of cerebral amyloid deposition should be monitored regularly, as these patients may be predisposed to develop cognitive dysfunction in the long‐term. Nevertheless, bearing in mind the limitations of the low availability and high cost of amyloid scans, future work should focus on investigating other more feasible surrogate markers, such as plasma biomarkers, for brain amyloid deposition.

Our study has several limitations. First, this was not a consecutive ICH cohort and therefore selection bias exists. Moreover, a substantial number of patients were lost to follow‐up, mainly due to personal reasons, which may therefore bias our results. In addition, the cognitive follow‐up protocol was not standardized, especially for the patients without cognitive symptoms who did not undergo regular cognitive assessments during their outpatient visits. Thus, these methods may have resulted in underestimation of the rate of cognitive decline. Second, cognitive change was determined using screening tools and the patients in this study did not undergo detailed neuropsychological assessments. Moreover, motor function, such as gait speed, was not assessed. Thus, this analysis could not evaluate whether changes in individual cognitive domains or motor functions are more specifically related to underlying SVD processes or regional amyloid deposition. The etiology (neurodegenerative or cerebrovascular process) underlying cognitive decline could not be accurately assessed, either. Third, a positive amyloid scan could indicate both parenchymal and vascular Aβ burden, and we were unable to clearly differentiate whether the increases in tracer retention were due to CAA, Alzheimer's disease, or age‐related amyloid pathology. In addition, tau‐mediated neurodegeneration was not assessed in the current study. The development of more PET tracers that specifically bind to vascular Aβ (Zhang et al., 2021) or a combined approach with tau imaging may help to define the respective contribution of these pathologies to cognitive decline in the future, as discussed previously. Fourth, the time window from the index ICH events to PiB scans was variable; therefore, our study cannot provide precise epidemiological data on post‐ICH cognitive impairment. As the focus of this study was to investigate the effects of amyloid in patients with hemorrhagic SVD, we believe that this limitation does not significantly affect our results. Lastly, the sample size in this study was relatively small and our findings should be mainly considered to be exploratory. However, this is the largest longitudinal ICH cohort with amyloid imaging reported to date, and we believe that our analysis provides an important indication of the significance of amyloid status on the cognitive outcomes of survivors of ICH.

In conclusion, we demonstrate an association between a positive amyloid scan and long‐term cognitive decline in patients who survive SVD‐related spontaneous ICH, which suggests that cerebral amyloid deposition is a potential driving factor for cognitive impairment in hemorrhagic SVD.

## AUTHOR CONTRIBUTIONS

*Conception and design*: Ya‐Chin Tsai, Hsin‐Hsi Tsai, and Ruoh‐Fang Yen. *Collection and assembly of data*: Ya‐Chin Tsai, Hsin‐Hsi Tsai, Chia‐Ju Liu, Sheng‐Sian Lin, Ya‐Fang Chen, Jiann‐Shing Jeng, Li‐Kai Tsai, and Ruoh‐Fang Yen. *Data analysis and interpretation*: Ya‐Chin Tsai and Hsin‐Hsi Tsai. *Final approval of manuscript*: Ya‐Chin Tsai, Hsin‐Hsi Tsai, Chia‐Ju Liu, Sheng‐Sian Lin, Ya‐Fang Chen, Jiann‐Shing Jeng, Li‐Kai Tsai, and Ruoh‐Fang Yen.

## CONFLICT OF INTEREST STATEMENT

The authors declare that there are no conflicts of interest that could be perceived as prejudicing the impartiality of the research reported.

### PEER REVIEW

The peer review history for this article is available at https://publons.com/publon/10.1002/brb3.3189.

## Supporting information

Supporting Information

## Data Availability

All data from this article are being held within National Taiwan University Hospital and will be shared with qualified investigators on request.
