# Supplementary material for: Cerebral amyloid deposition predicts long‐term cognitive decline in hemorrhagic small vessel disease
Source: Brain Behav. 2023 Aug 2;13(10):e3189. doi: 10.1002/brb3.3189 (PMC10570474; doi:10.1002/brb3.3189)
Supplement: Supplementary file 1 — Supporting Information [file BRB3-13-e3189-s001.docx]

| **Supplementary Table. Comparison of the demographics in patients with positive and negative amyloid scans.**   \|  \| **Included patients**  (*n* = 68) \| **Excluded patients**  (*n* = 77) \| ***P-*value** \| \| --- \| --- \| --- \| --- \| \| Male, % \| 45 (66.2%) \| 48 (62.3%) \| 0.729 \| \| **Age, years** \| **61.9 ± 12.0** \| **69.4 ± 11.1** \| **<0.001** \| \| **Years of education** \| **12.4 ± 4.0** \| **9.6 ± 4.0** \| **<0.001** \| \| Hypertension, % \| 59 (86.8%) \| 58 (75.3%) \| 0.095 \| \| Diabetes, % \| 11 (16.2%) \| 16 (20.8%) \| 0.527 \| \| Hypercholesterolemia, % \| 21 (30.9%) \| 17 (22.1%) \| 0.259 \| \| EGFR, mL/min \| 88.9 ± 23.6 \| 81.0 ± 26.4 \| 0.066 \| \| **MMSE** \| **27.2 ± 3.6** \| **20.9 ± 8.5** \| **<0.001** \| \| Lobar ICH \| 35 (51.5%) \| 48 (62.3%) \| 0.239 \| \| CAA \| 16 (23.5%) \| 29 (37.7%) \| 0.074 \| \| **Probable CAA** \| **11 (16.2%)** \| **27 (35.1%)** \| **0.013** \| \| **PiB PET(+)** \| **11 (16.2%)** \| **36 (46.8%)** \| **<0.001** \| \| Cerebral microbleed \|  \|  \|  \| \| Lobar CMBs (+) \| 44 (64.7%) \| 61 (79.2%) \| 0.063 \| \| **Number of lobar CMBs** \| **5.9 ± 13.5** \| **16.1 ± 25.8** \| **0.003** \| \| Deep CMBs (+) \| 35 (51.5%) \| 41 (53.2%) \| 0.869 \| \| Number of deep CMBs \| 3.9 ± 6.5 \| 3.7 ± 6.3 \| 0.815 \| \| **WMH volume, mL** \|  \|  \|  \| \| **Fazekas scale ≥ 2** \| **40 (58.8%)** \| **62 (88.3%)** \| **0.006** \| \| Volume, mL \| 14.6 ± 13.9 \| 20.3 ± 20.9 \| 0.061 \| \| **Lacunes, %** \| **30 (44.1%)** \| **48 (62.3%)** \| **0.031** \| \| MRI-visible enlarged perivascular spaces \|  \|  \|  \| \| Basal ganglia (> 20), % \| 34 (50.0%) \| 34 (44.2%) \| 0.509 \| \| **Centrum semiovale (> 20), %** \| **22 (32.4%)** \| **40 (58.8%)** \| **0.019** \| \| **Cortical superficial siderosis, %** \| **4 (5.9%)** \| **20 (26.0%)** \| **0.001** \| \| Hippocampal volume, mm^3^ \| 3760 ± 673 \| 3572 ± 664 \| 0.098 \|   Values are mean (± standard deviation) or number (percentage). Significant differences are shown in bold.  CAA: cerebral amyloid angiopathy, CMB: cerebral microbleeds; IQR: interquartile range; MMSE: Mini-Mental Status Exam; WMH: white matter hyperintensities. |
| --- | --- | --- | --- | --- | --- | --- | --- | --- | --- | --- | --- | --- | --- | --- | --- | --- | --- | --- | --- | --- | --- | --- | --- | --- | --- | --- | --- | --- | --- | --- | --- | --- | --- | --- | --- | --- | --- | --- | --- | --- | --- | --- | --- | --- | --- | --- | --- | --- | --- | --- | --- | --- | --- | --- | --- | --- | --- | --- | --- | --- | --- | --- | --- | --- | --- | --- | --- | --- | --- | --- | --- | --- | --- | --- | --- | --- | --- | --- | --- | --- | --- | --- | --- | --- | --- | --- | --- | --- | --- | --- | --- | --- | --- | --- | --- | --- | --- | --- | --- | --- | --- | --- | --- | --- | --- | --- | --- | --- |
